# Application of chromosome microarray analysis and karyotyping in diagnostic assessment of abnormal Down syndrome screening results

**DOI:** 10.1186/s12884-022-05139-3

**Published:** 2022-11-04

**Authors:** Han Kang, Lingxi Wang, Xingyu Li, Chonglan Gao, Yamei Xie, Yu Hu

**Affiliations:** Prenatal diagnosis department, Chengdu Women’s and Children’s Central Hospital, School of Medicine, University of Electronic Science and Technology of China, Chengdu, China

**Keywords:** Chromosome microarray analysis, Down syndrome screening, Karyotyping, Nuchal translucency thickness, Prenatal diagnosis

## Abstract

**Background:**

Down syndrome (DS) is the most common congenital cause of intellectual disability and also leads to numerous metabolic and structural problems. This study aims to explore the application value of chromosomal microarray analysis (CMA) and karyotyping in prenatal diagnosis for pregnant women with abnormal DS screening results.

**Methods:**

The study recruited 1452 pregnant women with abnormal DS screening results including 493 with an enlarged nuchal translucency thickness (NT ≥ 2.5 mm) and 959 with an abnormal second-trimester maternal serum biomarker screening results. They underwent amniocentesis to obtain amniotic fluid for CMA and karyotyping.

**Results:**

CMA identified 74/1452 abnormal results, which was more efficient than karyotyping (51/1452, *P* < 0.05.) CMA is equivalent to traditional karyotyping for identifying aneuploidies. Compared to karyotyping CMA identified 1.90% more copy number variants (CNVs) ranging from 159Kb to 6496Kb. However, 34.4% of them were recurrent pathogenic CNVs associated with risk of neurodevelopmental disorders. CMA identified 13 variants of uncertain significance (VUS) results and 1 maternal uniparental disomy (UPD) of chromosome 7. Karyotyping identified 3 mosaic sex chromosome aneuploidy and 4 balanced translocation which could not be identified by CMA. In enlarged NT group, karyotyping identified 80.9% abnormal results while in serum screening group karyotyping identified 35.7%. However, the incidence of pathogenic/likely pathogenic (P/LP) CNVs was nearly the same in both groups. That was because aneuploidies and gross duplication/deletion were previously screened out by NT scan.

**Conclusions:**

CMA and karyotyping have both advantages and disadvantages in prenatal diagnosis of pregnant women with abnormal DS screening results. However, there was not enough evidence to support routine CMA in pregnant women with abnormal DS screening results.

## Background

DS is the most common congenital cause of intellectual disability and also leads to numerous metabolic and structural problems. Since 1984 Irwin R and colleagues found an association between low maternal serum α-fetoprotein and fetal chromosomal abnormalities [1], several biomarkers have been observed in abnormally high or low concentrations in the serum of pregnant women whose fetuses are affected by DS [2–5]. Since 1990s, several studies have reported that enlarged NT at 10–14 weeks of gestation is associated with increased risk of trisomy 21 and other chromosomal defects [6–8]. Although screening for fetal aneuploidy with the use of cfDNA obtained from maternal plasma is highly effective [9], NT scan combined with biomarkers screening is in extensive use in economically underdeveloped areas and poor population. When screening tests predict a high risk of DS, an invasive diagnostic test (amniocentesis or chorionic villus sampling) is usually needed to confirm the diagnosis [10].

Before the era of microarray, G-banded karyotyping was the gold standard diagnostic test for pregnant women whose screening tests predict a high risk. The prevalence of congenital anomalies caused by pathogenic microdeletions and duplications is 1.2%, while the prevalence of congenital anomalies caused by common trisomies (trisomy 21, 18, 13) is only 0.2% [11]. For over a decade, CNV analysis by CMA has been broadly used for detection of genomic imbalances at a much higher resolution than conventional methods such as karyotyping. It was recommended as a first-tier approach for the prenatal evaluation of fetuses with structural anomalies observed by ultrasound [12, 13]. CMA includes array comparative genomic hybridization (aCGH) and single nucleotide polymorphism (SNP) array. Compared with aCGH, SNP array has the advantage of detecting triploidy and regions of homozygosity which might indicate UPD [14].

In this study we summarized the CMA and karyotyping results of 1452 cases with abnormal DS screening results.

## Methods

### Patients

**Fig. 1:**
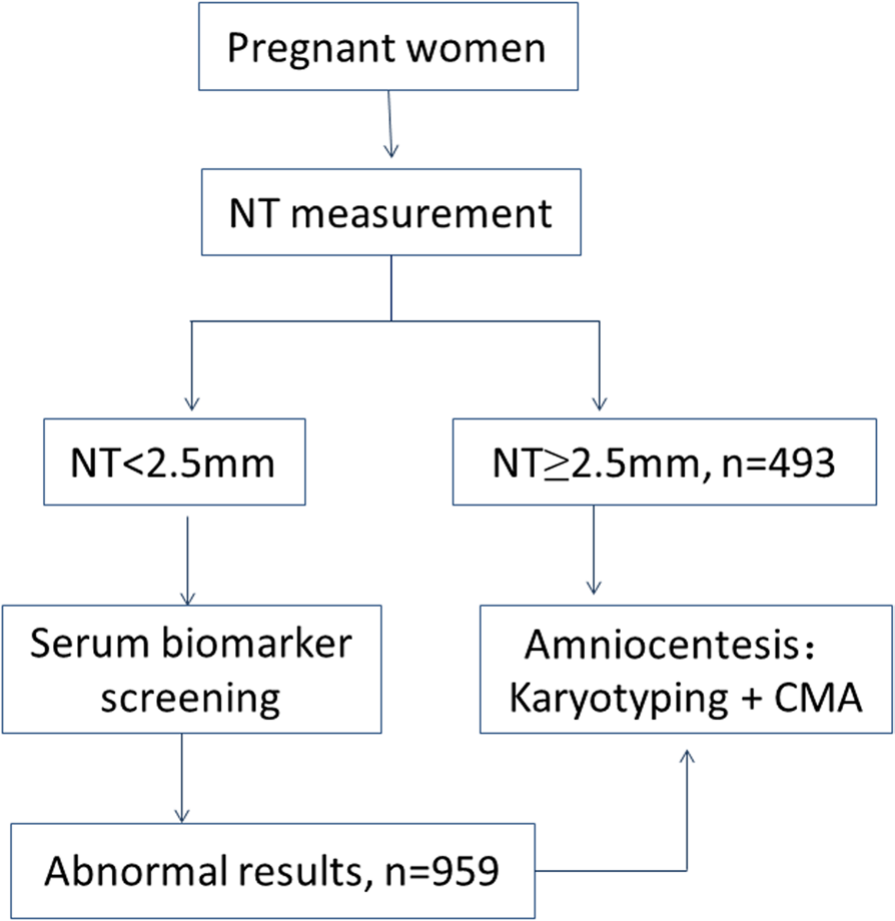
DS screening protocol in our department

DS screening protocol in our department is first-trimester ultrasound measures of NT combining with second-trimester maternal serum biochemical markers screening (Fig. 1). In this study, a total of 1452 pregnant women with abnormal DS screening results at the Chengdu Women’s and Children’s Central Hospital were enrolled in this study from January 2018 to November 2021: including 493 pregnant women with an enlarged NT measurement (NT ≥ 2.5 mm) and 959 with an abnormal maternal serum biomarker screening results (including high risk and borderline risk for trisomy 21, 18, high risk for open Neural Tube Defects (NTD), and abnormal multiple of median (MOM)). All these pregnant women underwent amniocentesis to obtain fetal amniotic fluid for SNP array and karyotyping. This study was approved by the Medical Ethics Committee of Chengdu Women’s and Children’s Central Hospital and all pregnant women signed informed consent forms.

### Second trimester biochemical markers screening

Free β human chorionic gonadotrophin (Free β-hCG) and α-fetoprotein (AFP) were quantitatively measured by AFP/F-β HCG Dual Kit on an automated time-resolved florescence immunoassay system TALENT-II analyzer (FENGHUA BIOLOGICAL, Guangzhou, China). The biomarker results were adjusted for maternal weight, maternal age, gestational week and Asian ethnicity, and the likelihood ratio of the fetus being affected with trisomy 21, trisomy 18 and NTD was calculated using PRSOFT software (FENGHUA BIOLOGICAL, Guangzhou, China). The patients were categorized as high-risk (> 1/270), borderline risk (1/271 ~ 1/1000), and low-risk (< 1/1001) for DS, high-risk (> 1/350), borderline risk (1/351 ~ 1/1000), and low-risk (< 1/1001) for trisomy 18, and an AFP MOM of greater than 2.5 for open NTDs.

### Karyotyping and CMA methodology

Three tubes (10 ml*3) of amniotic fluid were collected under ultrasound-guided localization at 18–25 gestational weeks. The first two tubes were used for karyotyping, and third one for CMA. For amniotic fluid samples with maternal cell contamination, the CMA test was performed after the amniotic fluid cells were cultured.

Karyotyping was performed independently by two individuals using two cell culture systems. After cell culture and sample preparation, a LABB M9120 instrument (Shanghai Beion Medical Technology, Shanghai, China) and matching image analysis software were used for chromosome karyotype scanning and analysis. At least three cell karyotypes were analyzed for each culture, and 20 karyotypes were counted. For the cases with chromosome mosaicism, more karyotypes were counted or analyzed. Karyotyping and descriptions were based on the International Human Cytogenomic Naming System (2020) [15].

Genomic DNA from amniotic fluid was extracted using a QIAamp DNA Blood Mini Kit (QIAGEN, Germany) according to the manufacturer’s instructions. An Affymetrix CytoScan 750 K Array (Affymetrix, USA) chip was used for CMA analysis according to the manufacturer’s instructions. ChAS 4.2 software was used for analysis.

### Results categorization

The detected results were categorized into aneuploidy and CNVs including gross deletion, gross duplication, submicroscopic deletion, submicroscopic duplication and loss of heterozygosity (ROH). An arbitrary line was drawn at 5 Mb to differentiate between submicroscopic and gross deletion and duplication.

### CNV interpretation and confirmation

All reported CNVs were based on the National Center for Biotechnology Information human genome build 38.The reported CNVs were classified by five-tiered system according to technical standards of the American College of Medical Genetics and Genomics (ACMG) [13] with the assistance of the following databases: the Database of Genomic Variants (DGV, http://dgv.tcag.ca/dgv/), the Online Mendelian Inheritance in Man database (OMIM, http://www.ncbi.nlm.nih.gov/omim), Clinical Genome Resourse (ClinGen, https://www.clinicalgenome.org/), ClinVar(https://www.ncbi.nlm.nih.gov/clinvar/),the Database of Chromosomal Imbalance and Phenotype in Humans using Ensembl Resources (DECIPHER, https://decipher.sanger.ac.uk/), and PubMed (https://www.ncbi.nlm.nih.gov/pubmed/). P, LP and VUS were shown in this study, while likely benign and benign were not.

### Inheritance studies

Inheritance studies were performed using Fluorescence in-situ Hybridization (FISH), conventional karyotyping, or CMA when necessary. For example, unbalanced translocation would need parents’ karyotyping or FISH depending on CNVs size.

### Statistical analysis

SPSS 19.0 software was used for statistical analysis. Comparisons between groups were performed using a χ2 test, and *P* < 0.05 was considered statistically significant.

## Results

### Overall result

In this study, a total of 1452 pregnant women were enrolled in: 493 had an enlarged NT, 959 were abnormal in serum screening. CMA and karyotyping findings were summarized in Table 1. In enlarged NT group CMA identified 46 abnormal results, karyotyping identified 39. In abnormal serum screening group, CMA identified 28 abnormal results, karyotyping identified 12. In total, CMA identified 74/1452 abnormal results, which was more efficient than karyotyping (51/1452, *P* < 0.05.) Especially in abnormal serum screening group, CMA identified more than twice abnormal results compared to karyotyping.

**Table 1:** Abnormal results of CMA and karyotype analysis in 1452 pregnant women with DS screening abnormalities

Group	Total	CMA results/case	Karyotype analysis results/case	*P*-value
Enlarged NT	493	46	39	> 0.05
Abnormal serum screening	959	28	12	< 0.05
Total	1452	74	51	< 0.05

### CMA results

CMA identified 74 abnormal results in total, with the size ranged from 159Kb deletion (microdeletion of the X chromosome, including Duchenne muscular dystrophy (DMD) gene) to 155 Mb whole chromosome gain/loss (XXX/X/XXY). They could be grouped into aneuploidy (42/1452), gross deletion/duplication (5/1452), submicroscopic deletion/duplication (27/1452). Besides, CMA identified 5 LOH and 13 VUS (Table 2). Overall, 34 P/LP CNVs were detected in 32 fetuses (Table 3, abnormal serum screening group: No 1–20, enlarged NT group: No 21–32): 2 case had a microdeletion in the region of azoospermia factor (AZF) locus of the Y chromosome, 3 case had microdeletion in the X chromosome involving DMD, Leri-Weill dyschondrostosis and ichthyosis respectively. 27 cases had an autosomal CNVs including 3 case of 1q21.1 recurrent microduplication syndrome, 1 case of 2p16.1p15 microduplication, 1 case of 3q12.1q12.3 microdeletion, 1 case of 3q29 microdeletion, 1 case of 4q35.1q35.2 microdeletion, 1case of Cri-Du-Chat syndrome and 9p24.3p21 duplication, 1 case of 7p22.3p21.3 duplication and 18p11.32q11.2 duplication, 1 case of 8p23.3p23.2 microduplication,1 case of 9p24.3q21.11 duplication, 1 case of 11q22.1q23.1 microduplication, 1 case of 11q24.3q25 microduplication, 1 case of 15q11.2 recurrent microdeletion syndrome, 1 case of 15q24.1q24.3 microduplication, 1 case of 15q15.2q15.3 microdeletion, 1 case of 16p13.11 recurrent microdeletion syndrome, 2 case of 16p13.11 recurrent microduplication syndrome, 1 case of recurrent 16p12.1 microdeletion syndrome, 1 case of 16p11.2 microduplication syndrome, 1 case of 16p13.3 microdeletion, 1 case of Smith-Magenis Syndrome, 1 case of 17p13.3p11.2 duplication, 1 case of renal cysts and diabetes (RCAD) syndrome, 1 case of 20q13.12q13.2 microduplication, and 1 case of 22q11 duplication syndrome. Among the 32 cases with P/LP CNVs identified by CMA mentioned above, only 5 cases (8, 17, 27, 29, 30) were identified by karyotyping.

**Table 2:** The findings of CMA in two groups of enlarged NT and serum screening

Categorization	Enlarge NT	Serum screening
Aneuploidy	34/6.9%	8/0.8%
Gross duplication/deletion	3/0.6%	2/0.2%
Microdeletion/duplication	9/1.8%	18/1.9%
LOH	3/0.6%	2/0.2%
VUS	5/1.0%	8/0.8%
Total	54	38

**Table 3:** P/LP CNVs in 32 fetuses by CMA

No	CMA results	Categorization	Known syndromes	Dosage sensitive gene/region	OMIM gene count	Size of CNVs/kb	Inheritance
1	arr[GRCh38]Yq11.22322658726_ 26274233 × 0	P	AZFc	/	11	3616	NA
2	arr[GRCh38] Yq11.223 (24889425_28231736)×0	P	AZFc	/	11	3342	father
3	arr[GRCh38]Xp21.1 (31809962_31968905)× 0	P	/	DMD	1	159	mother
4	arr[GRCh38]Xp22.316537109_8167604)×1	P	STS	STS/Xp22.31 recurrent region	4	1630	NA
5	arr[GRCh38]1q21.1q21.2(147053151_148360058)× 3	P	1q21.1 recurrent microduplication	1q21.1 recurrent region	9	1812	NA
6	arr[GRCh38]2p16.1p15(60148343_61784764)×3	LP	/	/	7	1636	de novo
7	arr[GRCh38]3q29 (193373606_195885016)×1	LP	/	/	16	2511	NA
8	arr[GRCh38]11q22.1q23.1102192300_111795977)× 3	LP	/	/	50	9606	de novo
9	arr[GRCh38]16p13.11 (14799119_16364567)×1	P	16p13.11 recurrent microdeletion	16p13.11 recurrent region	14	1565	mother (learning disorder)
10	arr[GRCh38]16p13.11p12.3 (15225421_18148856)× 3	P	16p13.11 recurrent microduplication	16p13.11 recurrent region	10	2923	mother
11	arr[GRCh38]16p12.1 (21728879_22430686)×1	p	Recurrent 16p12.1 microdeletion	/	5	702	father
12	arr[GRCh38]16p11.229401182_30178708)×3	P	16p11.2 microduplication syndrome	16p11.2 recurrent region	26	778	father
13	arr[GRCh38]17q12(36466620_37940921)×1	P	RCAD syndrome	HNF1B/17q12 recurrent (RCAD syndrome) region	14	1474	NA
14	arr[GRCh38]22q11.21(18153983_21110475)× 3	P	22q11 duplication syndrome	/	45	2956	mother
15	arr[GRCh38]11q24.3q25(130838148_132911316)×3	LP	/	/	2	2073	de novo
16	arr[GRCh38]1q21.1q21.2(145605589_149034959)× 3	P	TAR syndrome	1q21.1 recurrent region	27	3429	father
17–1	arr[GRCh38]7p22.3p21.3(43377_11114826)×3	P	/	/	62	11,071	NA
17–2	arr[GRCh38] 18p11.32q11.2(136227_23117390)×3	P	/	/	68	22,981	NA
18	arr[GRCh38] 1q21.1q21.2(146107656_149913567)×3	P	/	1q21.1 recurrent region	28	3806	mother
19	arr[GRCh38]15q11.2(22582283_23060000)×1	P	/	15q11.2 recurrent region	4	473	NA
20	arr[GRCh38]16p13.11(14799119_16433802)×3	P		16p13.11 recurrent region	14	1635	NA
21	arr[hg38]4q35.1q35.2(186098750–187,424,068)× 1	LP	/	/	5	1325	pat
22	arr[hg38] Xp22.33 or Yp11.32(490354–1,086,978 or 579,619–1,086,822)×1	P	Leri-Weill dyschondrostosis	SHOX	1	683	de novo
23	arr[GRCh38]15q24.1q24.3(74106238–77,930,504)×3	LP	/	/	40	3824	mother
24	arr[GRCh38]16p13.3(35,880 _270350)×1	P	α-Thalassemias	/	11	234	mother
25	arr[GRCh38] 15q15.2q15.3(43,209,432 _44249624)×1	LP	/	/	19	1040	de novo
26	arr[GRCh38]20q13.12q13.2(45,427,844 _51923590)×3	LP	/	/	49	6496	de novo
27	arr[GRCh38]17p13.3p11.2(150733_21615729)× 3	P	/	/	271	21,465	NA
28	arr[GRCh38]17p12p11.2(16003239_20644312)×1	P	Smith-Magenis Syndrome	FLCN、 RAI1	49	4641	NA
29	arr[GRCh38]9p24.3q21.11(208455_68398884)×3	P	/	/	177	68,190	NA
30–1	arr[GRCh38]5p15.33p14.3(113462_18666556)×1	P	Cri-Du-Chat syndrome	TRIO	55	18,553	NA
30–2	arr[GRCh38]9p24.3p21.3(208455_21487987)×3	P	/	/	78	21,280	NA
31	arr[GRCh38]8p23.3p23.2(2210719_3676067)×3	LP	/	/	1	1464	father
32	arr[GRCh38]3q12.1q12.3(99170215_102964080)×1(3.794 Mb)	LP	/	/	19	3794	NA

Case No 1–20: abnormal serum screening group, Case No 21–32: enlarged NT group

*DMD* Duchenne muscular dystrophy, *STS* Steroid sulphatase deficiency, *RCAD* renal cysts and diabetes, HNF1B hepatocyte nuclear factor 1beta, *NA* no inheritant result acquired, *TAR* Thrombocytopenia-absent radius, *SHOX* short stature homeobox, RAI1 retinoic acid induced 1, *TRIO* trio Rho guanine nucleotide exchange factor

CMA identified 13 VUS results, including 9 submicroscopic duplication and 4 submicroscopic deletion, with the size ranged from 840 kb–2411 kb. None of them was identified by karyotyping. CMA identified 5 ROH, including two cases involving chromosome 6 and 7 respectively. According to prenatal uniparental disomy (UPD) testing, the ROH of chromosome 7 was proved to be maternal UPD.

### Karyotype results

Traditional karyotype identified 52/1452 abnormal results (Table 4): including 32 trisomy 21 (No 1–32), 1 mosaic trisomy 21 (No 33), 3 trisomy 18 (No 34–36), 4 47,XXX syndrome (No 37–40), 1 Klinefelter syndrome (No 41), 1 Turner syndrome (No 42), 4 mosaic sex chromosome aneuploidy (No 43–46), 5 structural anomaly (No 47–52). Besides, 4 balanced translocation (No 53–56) and 1 mosaic balanced translocation (No 57) were identified. CMA identified all of these aneuploidies. However, CMA could not identified three of the four mosaic sex chromosome aneuploidy for their low proportion (<=10%). All of the 4 balanced translocation were inherited from healthy parents, and normal results of CMA also suggested they were truly balanced. For the mosaic balanced translocation, although CMA result was normal, we couldn’t discriminate it between truly balanced and unbalance.

**Table 4:** The findings of karyotyping in 1452 pregnant women

No	Karyotype results	Known syndromes	Inheritance
1–31	47,--,+ 21	Down syndrome	de novo/NA
32	46,--,rob(14;21)(q10q10),+ 21	Down syndrome	NA
33	mos 47,--,+ 21[39]/46,--[48]	Down syndrome(mosaic)	de novo
34–36	47,--,+ 18	Edwards syndrome	de novo
37–40	47,XXX	47,XXX syndrome	de novo
41	47,XXY	Klinefelter syndrome	NA
42	45,X	Turner syndrome	de novo
43	mos 45,X[61]/47,XXX[15]	Turner syndrome(mosaic)	de novo
44	mos 45,X [7]/46,XX[127]	Turner syndrome(mosaic)	de novo
45	mos 45,X [15]/46,XY[135]	Turner syndrome/Hermaphroditism	de novo
46	mos 47,XXX [13]/46,XX[140]	47,XXX syndrome(mosaic)	NA
47	46,--,dup(11)(q22.2q23.1)	/	de novo
48	46,--,der(4)t(4;17)(p16;p11.2)	/	father
49	46,--,del(17)(p11.2p11.2)	Smith-Magenis Syndrome	NA
50	mos 47,--,+psu idic(9)(q21.11) [13]/46,--[54]	/	NA
51	46,--,der(5)t(5;9)(p15.1;p22)	Cri-Du-Chat syndrome	father
52	47,--,t(7;18)(p21;q11.2),+mar	/	mother
53	45,--,rob(14;22)(q10;q10)	/	father
54	46,--,t(2;20)(p23;q13.1)	/	father
54	46,--,t(7;12)(q21.2;p13.1)	/	mother
56	46,--,t(17;20)(q21;q11.2)	/	mother
57	mos 46,--t(3;6)(q11.2;q25) [9]/46,--[43]	/	de novo

Case No 1–4, 32, 37, 38, 42, 44, 45, 47, 52, 53, 56, 57: abnormal serum screening group, the rest ones: enlarged NT group

### Discordant results between karyotyping and CMA

In this study, discordant results were observed in 29 cases between karyotyping and CMA: including 26 CNVs that was smaller than the detection limit of karyotyping (Table 3, No 1–7, 9–16,18-26,31–32), 3 mosaic sex chromosome aneuploidy with a mosaic fraction lower than 10% (Table 4, No 44–46).

## Discussion

CMA, also known as molecular karyotyping, has gradually replaced conventional G-banded karyotyping as the first-tier diagnostic test for the individual with developmental delay, intellectual disability, autism spectrum disorder, and/or multiple congenital anomalies, as well as for prenatal evaluation of fetuses with structural anomalies observed by ultrasound [12]. Compared with karyotyping, CMA is capable of detecting clinically significant submicroscopic aberrations up to a few kb. In this study, we used CMA (SNP array platform) and karyotyping for prenatal diagnosis of pregnant women with abnormal DS screening results. CMA is equivalent to traditional karyotyping for the prenatal diagnosis of aneuploidies. CMA provided additional clinically relevant information in 32 of pregnancies. In NT group, although CMA identified more abnormal cases than karyotyping, the difference was not statistically significant. However, in the serum screening group, there was statistically significant difference between CMA and karyotyping (*P* < 0.05). CMA could detect 1.8% more P/LP CNVs than karyotyping in NT group. The positive rate was lower than previous reports because the NT cut-off for invasive testing in our department is 2.5 mm vs. 3.0–3.5 mm in most previous studies. Consist with these studies, aneuploidy and gross deletion/duplication accounted for more than 80% chromosomal abnormalities (NT cut-off 2.5 ~ 3.0 mm: 80% ~ 90%, NT cut-off 3.5 mm: > 90%) [16–21]. CMA could detect 1.9% more CNVs than karyotyping in serum screening group, which is consistent with previous reports [22, 23]. Besides, we identified 2 mendelian monogenetic disease involving *DMD* gene and *HBA1* + *HBA2* gene respectively. Inheritance studies revealed the abnormalities was inherited from their mother.

Among the P/LP CNVs identified by CMA, 34.4% (11/32) were recurrent pathogenic CNVs associated with risk of neurodevelopmental disorders. In NT group no recurrent pathogenic CNVs was detected, while in serum group, 55.0% (11/20) were recurrent pathogenic CNVs. Whether there is an association between abnormal serum screening results and recurrent pathogenic CNVs requires further investigation. The penetrance for these recurrent pathogenic CNVs varies from race to race, [24, 25] and there was no large penetrance data available in Chinese population. So it was difficult to determine the clinical significance of these recurrent pathogenic CNVs, which would cause significant stress to pregnant women and their families, in some cases even resulted in the unnecessary abortion. According to previous reports, [25, 26], higher penetrance is seen with CNVs that have higher de novo frequencies. It was also reported that a strong association between IQ and the probability at which CNV deletions occur de novo [27]. Therefore inheritance studies of parents would be helpful to help determining source and counseling. Inheritance studies can bring some solace when the variant is inherited, or escalating of anxiety when it is de novo. However, inheritance of a variant from a healthy parent is no guarantee of it being benign—and the other way around. CMA identified 13 VUS, which is a difficult problem to genetic counseling. Inheritance studies of parents should be performed to help determining source and counseling. Pregnant women and their families should be fully informed of the possible outcomes and provide consent before CMA is performed.

The American College of Obstetrics and Gynecology (ACOG) and the American Maternal-Fetal Medicine Association’s 2016 guidelines clearly suggest CMA as a first-line prenatal diagnostic method in pregnant women with ultrasounds structural abnormalities [12, 28]. However, only a few reports had mentioned the effectiveness of CMA in pregnant women with DS screening abnormities [17–22]. In this study, CMA idenitified 1.9% more P/LP CNVs which is the first cause of congenital abnormities than karyotyping. However, the prevalence of P/LP CNVs in both groups (1.8 and 1.9%) is just a bit higher than that in common population (1.2%) [11]. What’s more, 34.4% of the P/LP CNVs detected were recurrent CNVs with uncertain outcome. So the necessity to perform CMA in pregnant women with DS screening abnormities remains indefinite. Multiple factors such as family history, pregnancy history, religious beliefs, ethical orientations and economic state should be considered.

When compared the incidence of different abnormities between NT and serum screening group, NT group had an obviously higher positive rate of aneuploidy. That was because a large part of aneuploidy was previously screened out by NT scan. In NT group, aneuploidy and gross deletion/duplication which could be identified by karyotyping accounted for 80.9% abnormal results (Table 2, (34 aneuploidy + 3 gross deletion/duplication + 1 microdeltion) / (34 aneuploidy + 3 gross deletion/duplication + 9 microdeltion/duplication + 1 UPD). Although the microdeletion/duplication accounted for a large part of abnormal results in serum screening group, the incidence between NT and serum screening group was almost the same, which was just a bit higher than that in common population [11]. This non-significant difference between women with abnormal DS screening results and common population would partly decrease the necessity of CMA in patients with abnormal DS screening results.

**Fig. 2:**
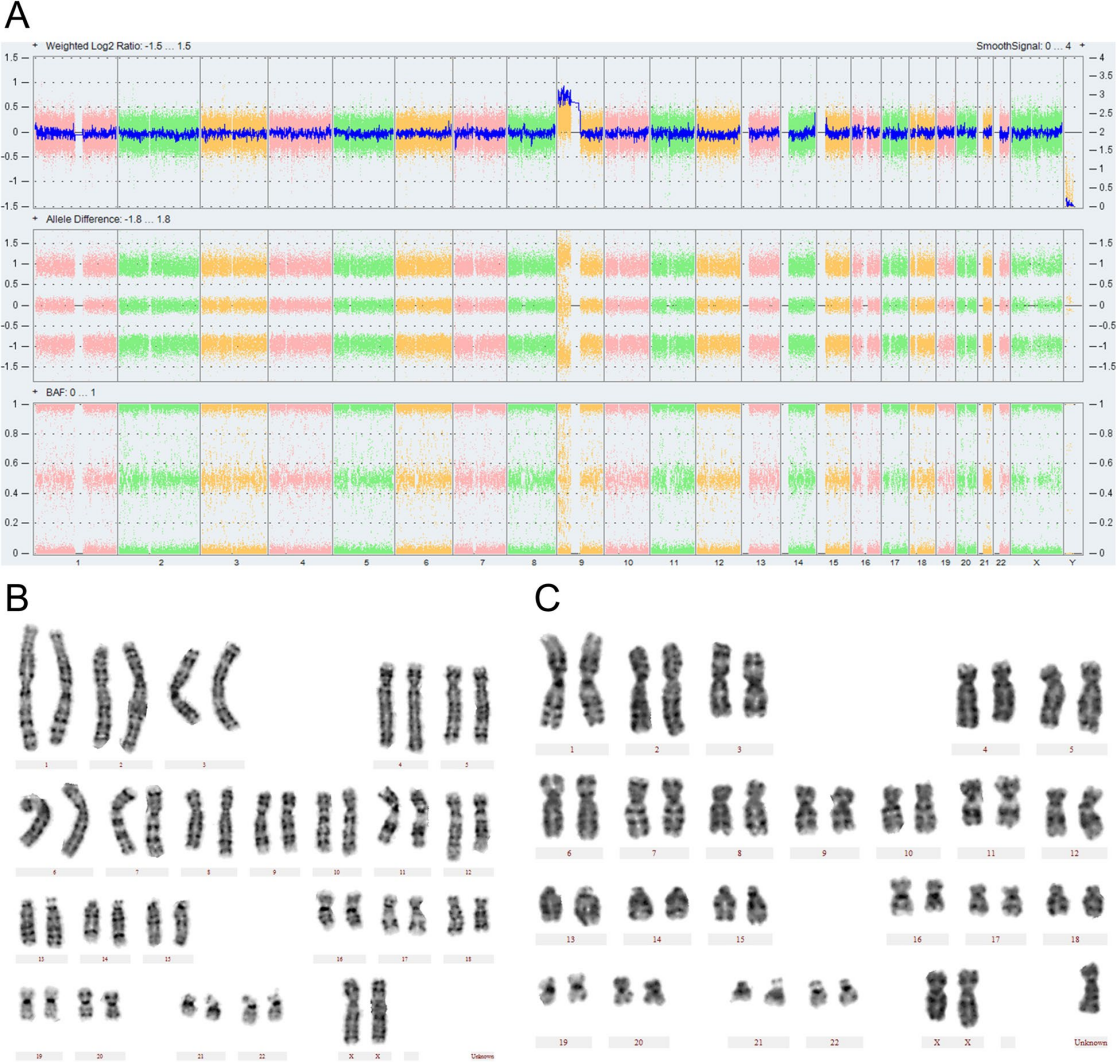
The CMA and karyotyping results of no 50 patients. **A**: CMA result, **B**: normal karyotype, **C**: abnormal karyotype

Despite the advantages of superior sensitivity and faster turn-around time, there are also some disadvantages compared to conventional karyotyping. CMA is unable to detect balanced chromosomal aberrations and mosaic chromosome abnormalities in low proportion. In this study, karyotyping identified 5 mosaic sex chromosome aneuploidy (Table 4, case 33, 43–46). CMA failed to identify three of them (Table 4, case 44–46). Although the proportions of abnormal cells were low, they might result in some symptoms of Turner syndrome or hermaphroditism according to previous reports [29, 30] and our experience in adults with such karyotype. In certain cases, karyotyping would give additional information for prognosis, such as case 43 and 50 in Table 4 and Fig. 2. In case 43 the karyotyping showed mos 45,X [61]/47,XXX[15] while CMA result was arr(X) × 1, and in case 50 the karyotyping showed mos 47,--,+psu idic (9)(q21.11)[13]/46,--[54] while CMA result was arr[GRCh38]9p24.3q21.11(208455_68398884)× 3. CMA can detect trisomy 13/21 but cannot discern whether it resulted from a non-disjunction event or due to a translocation. In such cases, karyotyping of the fetus and the parents is essential for determining reproductive risk for future offspring. Besides, karyotyping provided additional clinically relevant information in 5 pregnancies, 4 balanced translocation and 1 mosaic balanced translocation, which would be helpful for future pregnancies.

CMA and karyotyping have both advantages and disadvantages in prenatal diagnosis of pregnant women with abnormal DS screening results. So the genetic conseling before amniocentesis is very important, and both advantaged and disadvantages, charges of these tests should be carefully interpreted to parents so that they could make a choice whether CMA was performed.

## Conclusions

CMA and karyotyping have both advantages and disadvantages in prenatal diagnosis of pregnant women with abnormal DS screening results. However, there was not enough evidence to support routine CMA in pregnant women with abnormal DS screening results.

## Data Availability

The data that support the findings of this study are available on request from the corresponding author.

## List of abbreviations

CMA: Chromosomal microarray analysis
DS: Down syndrome
NT: Nuchal translucency thickness
CNVs: Copy number variants
VUS: Variants of uncertain significance
UPD: Uniparental disomy
P/LP: Pathogenic/likely pathogenic
aCGH: Array comparative genomic hybridization
SNP: Single nucleotide polymorphism
hCG: Human chorionic gonadotrophin
AFP: α-fetoprotein
NTD: Neural Tube Defects
MOM: Multiple of median
ROH: Loss of heterozygosity
ACMG: American College of Medical Genetics and Genomics
DGV: Database of Genomic Variants
OMIM: The Online Mendelian Inheritance in Man database
ClinGen: Clinical Genome Resourse
DECIPHER: The Database of Chromosomal Imbalance and Phenotype in Humans using Ensembl Resources
FISH: Fluorescence in-situ Hybridization
MOM: Multiple of median
DMD: Duchenne muscular dystrophy
AZF: Azoospermia factor
RCAD: Renal cysts and diabetes
ACOG: The American College of Obstetrics and Gynecology.
